# Notes on the genus *Psychostrophia* Butler, 1877 (Lepidoptera, Epicopeiidae), with description of a new species

**DOI:** 10.3897/zookeys.900.46973

**Published:** 2019-12-31

**Authors:** Si-Yao Huang, Min Wang, Xiao-Ling Fan

**Affiliations:** 1 Department of Entomology, College of Agriculture, South China Agricultural University, Guangzhou510642, Guangdong, China South China Agricultural University Guangzhou China

**Keywords:** cryptic species, East Asia, Geometroidea, oriental swallowtail moth, taxonomy

## Abstract

New information on the genus *Psychostrophia* Butler, 1877 is provided. A new species, *Psychostrophia micronymphidiaria* Huang & Wang, **sp. nov.**, is described from western, northern and northwestern Yunnan Province, southwestern China; it is similar to *P. nymphidiaria* (Oberthür, 1893) which is widely distributed in eastern, southern, western and central China. A new synonym is established: *Psychostrophia nymphidiaria* (Oberthür, 1893) (= *Stiboges lushanica* Chou & Yuan, 2001, **syn. nov.**). Some other taxonomic and nomenclatural notes on the genus are presented. A key to the species of the genus *Psychostrophia* is provided.

## Introduction

*Psychostrophia* Butler, 1877 is a small genus belonging to the family Epicopeiidae, which is widely distributed across Japan, China, and Indochina (Inoue 1992; Zhu et al. 2004; Owada 2011). This genus is characterized by the following characters: 1) hindwing with cilia mostly black, except for a white area between veins M1 and M3; 2) uncus long, thin, and tubular for most of its length; and 3) aedeagus with a cluster of slender cornuti, and coecum well developed and long (Minet 2003; Huang et al. 2019). Adults are diurnal, delicate moths, usually found flying along the forest edge or near water, visiting flowers or sucking nutrients from the damp ground. The immature stage is unknown for most of the members, and only the Japanese *P. melanargia* is found to feed on *Clethra barbinervis* of the family Clethraceae (Inoue 1982; Owada 2011). Until now only four species are known in this genus, viz. *P. melanargia* Butler, 1877, *P. nymphidiaria* (Oberthür, 1893), *P. picaria* Leech, 1897, and *P. endoi* Inoue, 1992, all of which have been previously recorded from China (Zhu et al. 2004; Huang et al. 2019).

Thus far, this genus has only been represented by *P. endoi* in Deqin County in Yunnan Province (Huang et al. 2019). The first observation of another member was of a worn-out male of a *Psychostrophia* species with a *P. nymphidiaria*-like appearance, flying together with another epicopeiid, *Burmeia leesi* Minet, 2003, at the edge of an evergreen broad-leaved forest near water at an altitude around 2550 m. This unexpected recording by the authors took place during a field survey conducted in Yaojiaping, Lushui County, on the western slope of the Gaoligong Mountains in western Yunnan province in the summer of 2018. Subsequently, the first author discovered in the Lepidoptera collection of the South China Agricultural University several males of this *P. nymphidiaria*-like species, which were collected from a vast area in Yunnan. Although at first glance they showed a striking similarity to *P. nymphidiaria* from other parts of China, some small but distinctive morphological differences were noticed. After examining the genitalia, these individuals were found to have distinguishing genital features, confirming them to represent a species distinct from the true *P. nymphidiaria*, making it the fifth species of the genus *Psychostrophia* Butler, 1877. It is described herein.

During the course of studying the genus *Psychostrophia*, the taxon *Stiboges lushanica* Chou & Yuan, 2001, originally described as a new butterfly, was found to be synonymous with *P. nymphidiaria* (Oberthür, 1893). Two names, P. melanargia ab. hemimelaena Seitz, 1912 and P. melanargia ab. catenifer Seitz, 1912 are unavailable as infrasubspecific, even after Zhu et al. (2004) provided a description of P. melanargia ab. hemimelaena. All the synonymic relationships and unavailable names mentioned above are discussed in detail below.

## Material and methods

Specimens examined in this study were all collected in daytime using an insect net and subsequently deposited in the collection of South China Agricultural University (**SCAU**), Guangzhou. The photographs of the holotype of *S. lushanica* in the collection of Northwest Agriculture and Forestry University (**NWAFU**) were provided courtesy of Dr Guo-xi Xue and used here under permission of Dr Xiangqun Yuan. Photographs of all adult specimens examined were taken using a Nikon CoolPix S7000 camera and the habitat photographs with a Sony DSC-RX100 v1.00 camera. Abdomens were removed and macerated in 10% NaOH for examination of genitalia. Photographs of genitalia of *Psychostrophia* spp. were taken under a Keyence VHX-5000 digital microscope. Adult and genitalia photographs were all processed using Adobe Photoshop CS5 software. Terminology for adults and genitalia follows Klots (1970) and Minet (2003). The specimen code for linking adult and genitalia together is numbered from PSY001 to PSY017.

## Taxonomy

### Genus Psychostrophia

Taxon classificationAnimaliaLepidopteraEpicopeiidae

Butler, 1877

710D2439-165E-5D97-AB4F-444D33E9A84E

Psychostrophia Butler 1877: 401.

#### Type species.

*Psychostrophia melanargia* Butler, 1877 (Yokohama, Japan).

### Psychostrophia micronymphidiaria

Taxon classificationAnimaliaLepidopteraEpicopeiidae

Huang & Wang sp. nov.

A389E044-D0AE-5510-94C4-5C9CEBB0BEBD

http://zoobank.org/AF2ECA37-9EE3-4EDD-A074-5BA0766976E6

Figs 1–4 , 9 , 10

Psychostrophia nymphidiaria : Huang et al. 2019: 40 [misidentification].

#### Type material.

***Holotype***: male, altitude 2779–2927 m, 27.V.2016, near Shajiama Bridge, Tacheng Town, Weixi Lisu Autonomous County, Diqing Tibetan Autonomous Prefecture, Yunnan Province, leg. Zhen-fu Huang, Qi-tong Huang and Jing Tang, PSY001. ***Paratypes***: 1 male, same label as holotype, PSY002; 1 male, altitude 2550 m, 15.VII.2018, Yaojiaping, Lushui County, Nujiang Lisu Autonomous Prefecture, Yunnan Province, PR China, leg. Si-yao Huang, PSY003; 1 male, 6.VII.2014, altitude 2900–3000 m, Mt Diancang, Dali Bai Autonomous Prefecture, Yunnan Province, leg. Hao Huang, PSY004; 1 male, altitude 2850 m, 7.VII.2013, Tacheng Town, Weixi Lisu Autonomous County, Diqing Tibetan Autonomous Prefecture, Yunnan Province, leg. Zhen-fu Huang, Hai-ling Zhuang and Min Wang, PSY005. The type series is deposited in the Insect Collection of Department of Entomology, South China Agricultural University (SCAU), Guangzhou, P. R. China.

#### Diagnosis.

Externally, *P. micronymphidiaria* sp. nov. is characterized and distinguished from its closest relative, *P. nymphidiaria* by a smaller size (length of forewing 16–17 mm vs 18–22 mm in *P. nymphidiaria*), more slender discal cell bar with the tip pointing to the tornus (in *P. nymphidiaria* the discal cell bar is robust and short, the tip shifting basally and pointing to the dorsum), and a narrower costal black border on the dorsal forewing. The male genitalia of *P. micronymphidiaria* sp. nov. can be distinguished from those of *P. nymphidiaria* by the following points: 1) the juxta is much narrower and more strongly sclerotized, while it is much broader and more membranous in *P. nymphidiaria*; 2) the valva has a narrower praesacculus, while it is broader in *P. nymphidiaria*; 3) the aedeagus is longer than the coecum, while it is shorter than the coecum or equal to it in *P. nymphidiaria*; 4) coecum and aedeagus are more sclerotized, while they are more membranous in *P. nymphidiaria*.

#### Description.

**Figures 1–8.**
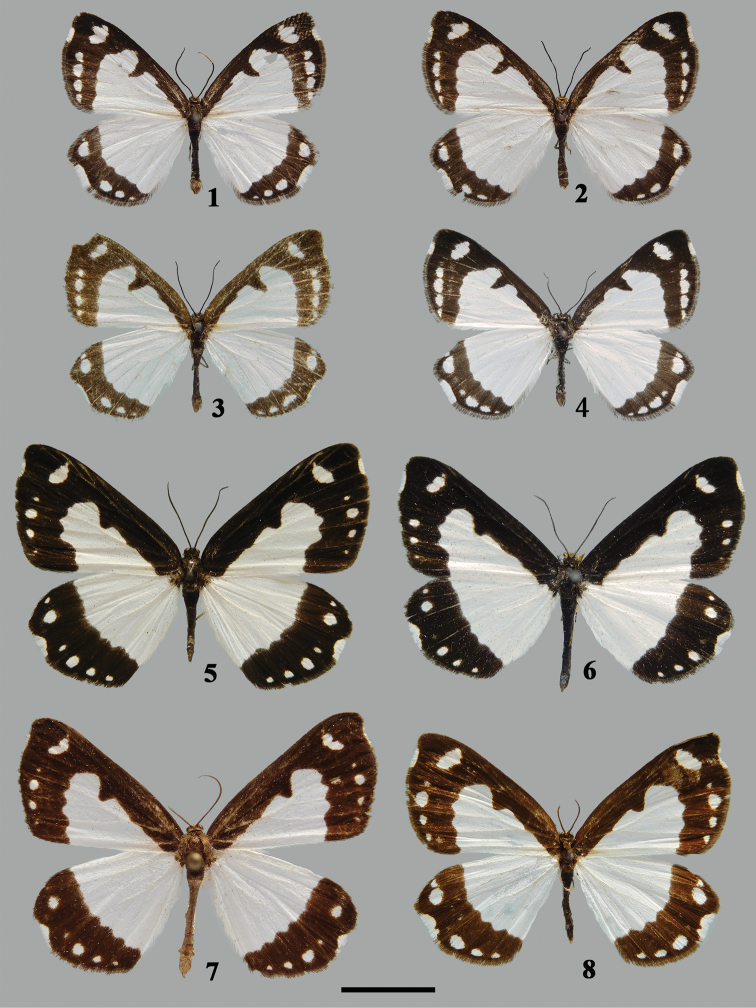
Males of *Psychostrophia* spp. **1***Psychostrophia micronymphidiaria* sp. nov., holotype, Weixi, Yunnan, PSY001 **2** ditto, paratype, Weixi, Yunnan, PSY002 **3** ditto, paratype, Lushui, Yunnan, PSY003 **4** ditto, paratype, Dali, Yunnan, PSY004 **5***Psychostrophia nymphidiaria*, Jiangshan, Zhejiang, PSY006 **6** ditto, Qingyuan, Zhejiang, PSY008 **7** ditto, Nanling, Guangdong, PSY009 **8** ditto, Yingjing, Sichuan, PSY007. Scale bar: 1 cm.

**Male** (Figs 1–4). Forewing length 16–17 mm (*n* = 5). Head black; antenna black, filiform. Thorax and abdomen black dorsally. Forewing ground color black with well-developed white patterns. White triangular zone extending from wing base to postmedial area, ending in wavy edge; cell bar at end of discal cell slender; subapical area with oval white patch, center sometimes extending outwards. Submarginal series comprising four white spots extending from vein M_2_ to anal angle. Cilia black from apex to vein R_5_, white from R_5_ to middle portion of cell M_1_, becoming black again from medial portion of cell M_1_ to tornus; sometimes cilia white only between vein R_5_ and vein M_1_. Dorsally, hindwing ground color white at inner two-thirds and black at outer one-third, junction line between white and black area wavy; submarginal series consisting of four to six white spots of different sizes, extending from apex to tornus; cilia black from apex to vein M_1_, white from M_1_ to medial portion of cell M_2_, becoming black again from medial portion of cell M_2_ to tornus.

**Figures 9–14.**
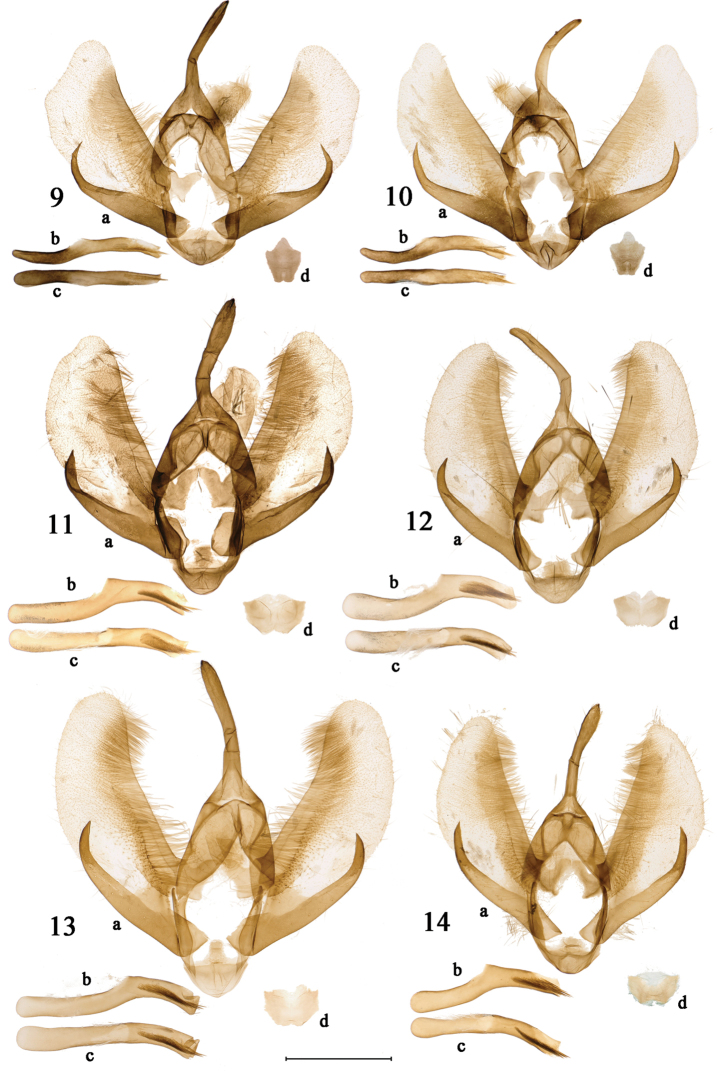
Male genitalia of *Psychostrophia* spp. **9***Psychostrophia micronymphidiaria* sp. nov., holotype, Weixi, Yunnan, PSY001 **10** ditto, paratype, Weixi, Yunnan, PSY002 **11***Psychostrophia nymphidiaria*, Jiangshan, Zhejiang, PSY006 **12** ditto, Qingyuan, Zhejiang, PSY008 **13** ditto, Nanling, Guangdong, PSY009 **14** ditto, Yingjing, Sichuan, PSY007. **a** = male genitalia capsule with juxta removed; **b** = aedeagus lateral view; **c** = aedeagus dorsal view; **d** = juxta. Scale bar: 1 mm.

***Male genitalia*** (Figs 9, 10). Uncus tubular, relatively long, and slender. Tegumen broadly U-shaped in ventral view, rather short and broad. Subscaphium moderately sclerotized, bearing setae in ventral and distal areas. Costula at base of costa, consisting of two sclerotized, crescent-shaped processes connected by a membrane. Juxta small and shield-like, strongly sclerotized. Saccus sclerotized, short and diamond-shaped. Valva shape varies from broad and stout to relatively slender, inner surface densely setose. Costa strongly sclerotized. Sacculus strongly sclerotized, broadened basally, narrowing distally. Praesacculus strongly sclerotized and bending upwards, ending with long and sharp tip. Aedeagus long and slender, sclerotized, cluster of long and thin cornuti present distally. Coecum strongly sclerotized, slightly shorter than aedeagus.

**Female.** Unknown at present.

#### Distribution.

This species is currently known to occur in western, northern and northwestern Yunnan province of China (Fig. 25).

#### Etymology.

The specific name *micronymphidiaria* is the combination of prefix *micro*- and *nymphidiaria*, referring to the size of the new species, which is smaller than *P. nymphidiaria*.

#### Bionomics.

This species has been found to fly at the periphery of evergreen broad-leaf forests or conifer-broadleaf forests near water, at altitudes above 2500 m (Figs 21, 22) from late May to mid July. Adults are diurnal and commonly found flying at a slow pace above bushes.

#### Remarks.

At present this species is restricted to habitats at altitudes above 2500 m in the Yunnan Province of southwestern China. Conversely, *P. nymphidiaria* is distributed across a vast area ranging from Sichuan Province to Zhejiang Province and extending southwards to northern Guangdong Province, typically preferring habitats where the altitude does not exceed 2200 m (usually from 300 to 2000 m).

### Psychostrophia nymphidiaria

Taxon classificationAnimaliaLepidopteraEpicopeiidae

(Oberthür, 1893)

5882313B-415B-5BB3-B095-920A94DF30F9

Figs 5–8 , 11–14 , 15

Abraxas nymphidiaria Oberthür 1893: 34, pl. 2, fig. 28. [Type locality: “Rencontrée pendant le voyage de Ta-Tsien-Lou à Mou-Pin” (Road from Kangding to Baoxing)].
Psychostrophia nymphidiaria (Oberthür): Leech 1897: 189; Seitz 1912: 278; Minet 2003: 473, 479, fig. 4.
Stiboges lushanica Chou and Yuan 2001: 142, fig. 15, 16. syn. nov. (Riodinidae).

#### Material examined.

Photos of holotype of *Stiboges lushanica*, male, printed label in Chinese “[Sichuan, Lushan, leg. Bing-hong Wang]”/ printed red label “Holotype” / red label “*Stiboges lushanica* Chou et etc., IDENT. IO CHOU” (NWAFU); 1 male, 22.VI.2003, Nanling Mts, Guangdong Province, leg. Min Wang, PSY009 (SCAU); 1 female, 2.VIII.2003, Huanjiang Maonan Autonomous County, Guangxi Zhuang Autonomous Prefecture, leg. Min Wang (SCAU); 1 male, 10.V.2018, Luding County, Sichuan Province, leg. Min Wang (SCAU); 1 male, altitude 1700–1900 m, 13.VI.2012, Mt Niba, Yingjing County, Ya’an City, Sichuan Province, leg. Xiao-hua Deng & Hou-shuai Wang, PSY007 (SCAU); 1 male, 11.VIII.2016, Shuangxikou Town, Jinyun County, Jiangshan City, Zhejiang Province, leg. Shu-qin Ji & Hou-shuai Wang, PSY006 (SCAU); 1 male, 25.VII–15.VIII.2018, Qingyuan County, Lishui City, Zhejiang Province, leg. Qing-song Wu, PSY008 (SCAU); 1 male, altitude 1500 m, 3. VI. 2019, Guanmenshan, Shennongjia, Yichang City, Hubei Province (SCAU); 2 males, 21.V.2011, Huanggangshan, Mt Wuyi, Fujian Province, leg. Zhen-fu Huang & Qi-tong Huang (SCAU); 2 males, altitude 1300 m, 13.VIII.2014, Mt Tianping, Zhangjiajie City, Hunan Province, leg. Lan-lan Huang, Wan Lu, Qi-tong Huang & Min Wang (SCAU).

#### Remarks.

Taxon *Stiboges lushanica* Chou & Yuan, 2001 was described based on two specimens taken in Lushan County, Ya’an City in western Sichuan Province. Kishida (2006) was first to point out that this “butterfly” taxon is conspecific with *P. nymphidiaria*, but did not synonymize it formally. With the help of Dr Guo-xi Xue and the permission from Dr Xiangqun Yuan, the photographs of the holotype could be examined. The holotype is a male of the oriental swallowtail moth species *P. nymphidiaria*. Although the male genitalia were not illustrated, the description by Chou and Yuan (2001) as well as the photographs suggest that these individuals are undoubtedly conspecific with this epicopeiid moth species commonly found in that area, rather than a bona species of the riodinid butterfly genus *Stiboges* Butler, 1876. Although their mimicry relationship makes them morphologically similar, one can easily recognize this moth species simply by the filiform antenna. Thus, *S. lushanica* is considered a junior synonym of *P. nymphidiaria* (syn. nov.).

Nevertheless, according to Dr Guo-xi Xue and Dr Xiangqun Yuan, there is another specimen also bearing the holotype red label of *S. lushanica* in the collection of NWAFU, and this specimen is illustrated here for the first time (Fig. 16). The information on the labels is interpreted as follows: “[Sichuan, Lushan, leg. Jing-hua Wang]”/ printed red label “Holotype” / red label “芦山白蚬蝶, *Stiboges lushanica* Chou et Yuan, IDENT. IO CHOU”. This specimen is a female of *Stiboges elodinia* Fruhstorfer, 1914 in the opinion of Callaghan (2009). Chou and Yuan (2001) stated in English that the sex of the holotype of *S. lushanica* was female, which would suggest that this taxon is a true butterfly. However, the descriptions in both Chinese and English have all clearly expressed that the holotype is conspecific with the male of *P. nymphidiaria* and definitely not a female riodinid butterfly. According to Article 73.1.1 of the Code (ICZN 1999), if an author, when establishing a new nominal species-group taxon, states in the original publication that one specimen, and only one, is the holotype, or “the type”, or uses some equivalent expression, that specimen is the holotype fixed by original designation. The specimen of *P. nymphidiaria* pictured in figure 16 of Chou and Yuan (2001) was fixed as the holotype of *S. lushanica* since the word “holotype” was plainly used in Chinese in the legend under this figure. The holotype status of that female riodinid butterfly, which was subsequently labeled as the holotype of *S. lushanica*, is therefore invalid, making the true holotype of this taxon the specimen of *P. nymphidiaria* shown in figure 16 of Chou and Yuan (2001).

During the study of the populations of *P. nymphidiaria* from various localities, it was found that males vary externally and in their genitalia. The median white zone on the dorsal forewing varies in shape and size, and the genitalia vary in the shape of the valva, length and width of the praesacculus, and the ratio of the aedeagus to the coecum (Figs 5–8, 11–14). It is possible that cryptic species still exist within *P. nymphidiaria* sensu lato, a possibility that deserves a more careful investigation including the examination of more adults and genitalia of both sexes, as well as conducting DNA barcoding in the future.

#### Distribution.

China (Sichuan, Hubei, Hunan, Zhejiang, Fujian, Guangdong, Guangxi Zhuang Autonomous Region) (Fig. 25)

**Figures 15–18.**
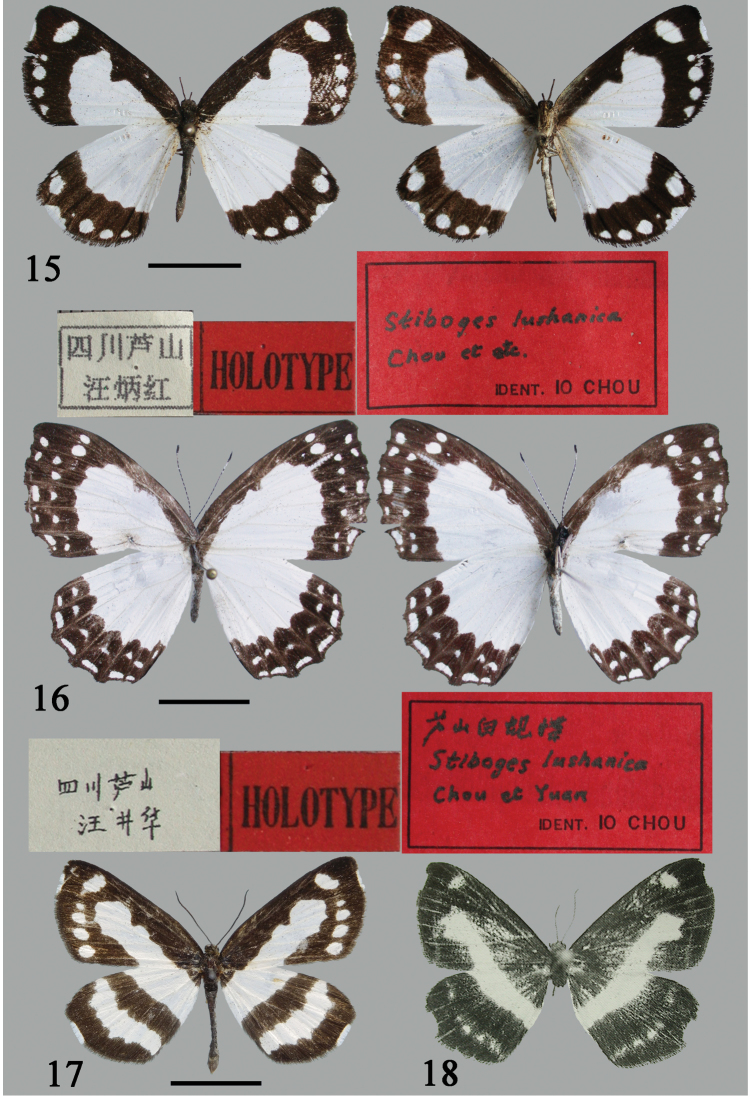
Adults of *Psychostrophia* spp. and *Stiboges* sp. **15, 17–18** male **16** female: **15***Psychostrophia nymphidiaria*, holotype of *Stiboges lushanica*, Lushan, Sichuan **16***Stiboges elodinia*, mislabeled specimen of “holotype” of *Stiboges lushanica*, Lushan, Sichuan **17***Psychostrophia picaria*, Shennongjia, Hubei, PSY017 **18** holotype of *Psychostrophia endoi*, Xam Neua, Laos, from Inoue (1992). Scale bars: 1 cm.

### Psychostrophia picaria

Taxon classificationAnimaliaLepidopteraEpicopeiidae

Leech, 1897

4E574A1F-8F97-5E60-B849-F747B1197DEF

Figs 17 , 19

Psychostrophia picaria Leech 1897: 189, pl. VI, fig. 11. [Type locality: Changyang, Ichang (now Yichang), Central China]; Seitz 1912: 278; Minet 2003: 473, 478, fig. 5, 24.

#### Material examined.

1 male, altitude 1000–1400 m, 13.V.2015, Muyu Town, Shennongjia, Yichang City, Hubei Province, leg. Yu-fei Li, PSY017 (SCAU); 1 female, 11–14.V.2007, Mt Tianping, Zhangjiajie City, Hunan Province, leg. Liu-sheng Chen, Zhen Li & Yang Long (SCAU); 3 males, 9.VII.2015, Mt Simian, Chongqing City, leg. Si-yao Huang (SCAU); 1 male, 1 female, 1.VII.2003, Mt Maoer, Guangxi Zhuang Autonomous Region, leg. Min Wang (SCAU).

#### Remarks.

Huang et al. (2019) stated that the difference in male genitalia between *P. endoi* and *P. picaria* lies in the shape of the valva, which protrudes more at the apex in *P. endoi*. However, the more protruding valva apex is actually found in *P. picaria*. The photographs of adult and male genitalia of a *P. picaria* collected in Shennongjia, Yichang, Hubei Province have been illustrated here for comparison. Judging from the structures of the male genitalia, both the sacculus and praesacculus of *P. picaria* are thicker than those of *P. endoi*, and the upper lobe of the juxta is much broader and longer than that of *P. endoi*. However, given that the valva structure is variable in *P. nymphidiaria*, it is possible that the differences in valva structure mentioned above between these two species are still not constant. Thus, only the shape of the juxta can currently be regarded as a true distinguishing characteristic. More material of male *P. endoi* should be examined to confirm such differences.

#### Distribution.

China (Hubei, Hunan, Chongqing, Guangxi Zhuang Autonomous Region) (Fig. 25).

**Figures 19, 20.**
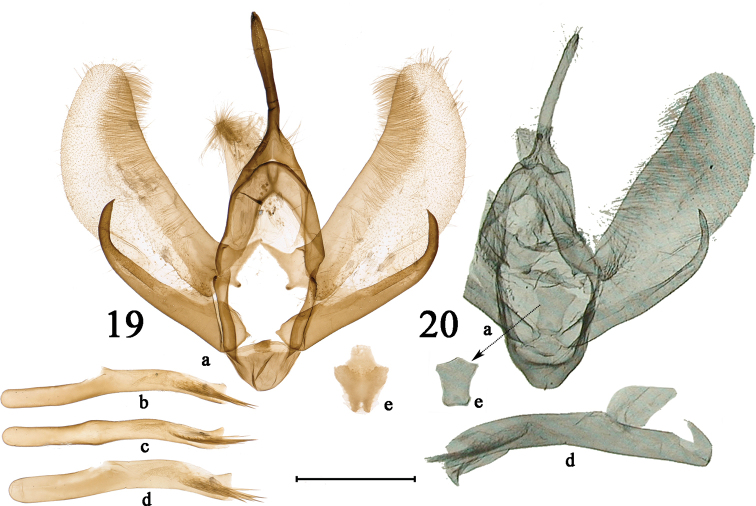
Male genitalia of *Psychostrophia* spp. **19***Psychostrophia picaria*, Shennongjia, Hubei, PSY017 **20** holotype of *Psychostrophia endoi*, Xam Neua, Laos, from Inoue (1992). **a** = male genitalia capsule with juxta removed; **b** = aedeagus lateral view; **c** = aedeagus dorsal view; **d** = flattened aedeagus in lateral view; **e** = juxta. Scale bar: 1 mm (**19**).

### Psychostrophia endoi

Taxon classificationAnimaliaLepidopteraEpicopeiidae

Inoue, 1992

E9432F1A-F6F2-5FF3-8AC3-262F7FAD1FDF

Figs 18 , 20

Psychostrophia endoi Inoue 1992: 149, figs 1, 2. [Type locality: Sam Neua (Xam Neua), Laos]; Huang et al. 2019: 44, figs 25–29.

#### Remarks.

The adult and genitalia figures of the holotype from Inoue (1992) have been reproduced for comparison.

#### Distribution.

China (Yunnan, Guizhou, Guangxi Zhuang Autonomous Region), Laos (Xam Neua) (Fig. 25).

### Psychostrophia melanargia

Taxon classificationAnimaliaLepidopteraEpicopeiidae

Butler, 1877

9FCE9F8E-7E52-53C8-80A0-17FF19E249F5

Psychostrophia melanargia Butler 1877: 401. [Type locality: Yokohama, Japan]; Leech 1897: 189; Seitz 1912: 278, pl. 48, line f; Minet 2003: 473, fig. 3; Zhu et al. 2004: 224, fig. 156, pl. VI, fig. 3.
Psychostrophia hemimelaena Seitz, 1913 [sic]: Zhu et al. 2004: 225, fig. 157, pl. VI, fig. 4.

#### Remarks.

Zhu et al. (2004) recorded *P. melanargia* Butler, 1877 and *P. hemimelaena* Seitz, 1913 (sic), which was originally described as an aberration of *P. melanargia*, viz. P. melanargia ab. hemimelaena Seitz, 1912, from Dailing, Heilongjiang Province and Mt Changbai, Jilin Province, respectively. According to Article 45.6.2 of the Code (ICZN 1999), the name *hemimelaena* as well as the name *catenifer* Seitz, 1912, which was also published as a new aberration, are invalid because they are infrasubspecific due to the use of the term “ab.” when described. Although these two names were subsequently regarded as P. melanargia var. hemimelaena and P. melanargia var. catenifera (sic) in the catalogue by Dalla Torre (1924), this action is at most an “elevation in rank” because no description and definition of these taxa can be traced throughout the catalogue. According to Article 45.5.1 of the Code (ICZN 1999), an infrasubspecific name cannot be made available from its original publication by any subsequent action (such as “elevation in rank”) except by a ruling of the Commission. Thus, the name P. melanargia ab. catenifer Seitz, 1912 is still unavailable. It should also be clarified that the correct spelling of this aberration is *catenifer*, not *catenifera*, as indicated in Dalla Torre (1924) and Beccaloni et al. (2003).

The matter of the name P. melanargia ab. hemimelaena is more complicated. As already mentioned above, according to Article 45.5.1 of the Code (ICZN 1999), a name that has infrasubspecific rank under the provisions of this Article cannot be made available from its original publication by any subsequent action (such as “elevation in rank”) except by a ruling of the Commission. Article 45.5.1 also states that when a subsequent author applies the same word to a species or subspecies in a manner that makes it an available name (Articles 11–18), even if he or she attributes authorship of the name to the author of its publication as an infrasubspecific name, that subsequent author thereby establishes a new name with its own authorship and date. The name *hemimelaena* seemed to have been made available under Article 45.5.1 by Zhu et al. (2004) as *Psychostrophia hemimelaena* Zhu, Wang & Han, 2004 because they gave a description in Chinese. This would mean that Zhu, Wang and Han 2004 published a new name with its own authorship and date. However, according to Dr Gerardo Lamas (pers. comm.), the actions of Zhu et al. (2004) did not actually comply with Articles 13.1.1, 16.4.1 and 16.4.2 (ICZN 1999). They require every new specific and subspecific name published after 1999, except a new replacement name (a nomen novum), for which the name-bearing type of the nominal taxon it denotes to is fixed automatically (Art. 72.7), must also be accompanied in the original publication by a description or definition that states in words characters that are purported to differentiate the taxon (Article 13.1.1), by the explicit fixation of a holotype, or syntypes, for the nominal taxon (Article 16.4.1) and where the holotype or syntypes are extant specimens, must be a statement of intent that they will be (or are) deposited in a collection and a statement indicating the name and location of that collection (Article 16.4.2). Zhu et al. (2004) did not differentiate *hemimelaena* from any other taxon in the genus *Psychostrophia* in their description, fix a holotype for the name *hemimelaena*, nor state where the “holotype” was because their actions were not deliberate, nor did they intend to make the name *hemimelaena* available. Thus, the name Psychostrophia melanargia ab. hemimelaena Seitz, 1912 is still unavailable, bearing the original authorship and date.

It is worth noting that the year of publication of P. melanargia ab. hemimelaena is 1912, not 1913 as indicated by Dalla Torre (1924), Beccaloni et al. (2003) and Zhu et al. (2004). This name had been published on page 278 in the section on the Uraniidae written by A. Seitz. Although no information on date can be traced throughout the whole section, according to Griffin (1936), the text of the Uraniidae section in the German version of Seitz (1912) was published in Lieferung 99 and received at the British Museum of Natural History on 25.VI.1912. Part 99 encompasses pages 265–344 and plates 49 and 53. The figure of P. melanargia ab. hemimelaena first appeared on plate 48 published in Lieferung 100, which was received at the British Museum of Natural History on 13.VIII.1912. Part 100 encompasses pages 345–392 and plates 48 and 50. Thus, the publication date of this name must be earlier than 25.VI.1912 and definitely not 1913.

The presence of *P. melanargia* in northeast China is still debatable. Its only known host plant, *Clethra barbinervis*, the Japanese sweet shrub, is distributed across Japan, Korea, and South and East China. The northernmost distribution record in China is from Mt Lao in the Shandong Province, and this plant is not currently recorded in the flora of northeast China (Qin and Fritsch 2005). Moreover, the specimen figured in Zhu et al. (2004) is not significantly different from the individuals commonly found in Japan, which suggests that the individuals examined by Zhu et al. (2004) were collected from somewhere within the geographic range of *P. melanargia* in Japan and mislabeled as being collected in northeast China. Thus, it is unlikely that the geographic range of *P. melanargia* extends to northeast China, and this species should therefore be excluded from the Chinese fauna. There is a similar case in Lepidoptera regarding the distribution of *Neope niphonica* Butler, 1881. Takahashi (1996) concluded that an individual of *N. niphonica* labeled as “Kirin, Manchoukuo, 1941-VII-17 (now Jilin Province, PR China)” had been mislabeled, because the host plant genus of this butterfly was not found in northeast China and the wing pattern did not differ from populations found in central Japan.

#### Distribution.

Japan (Honshu, Shikoku, Kyushu) (Fig. 25).

**Figures 21, 22.**
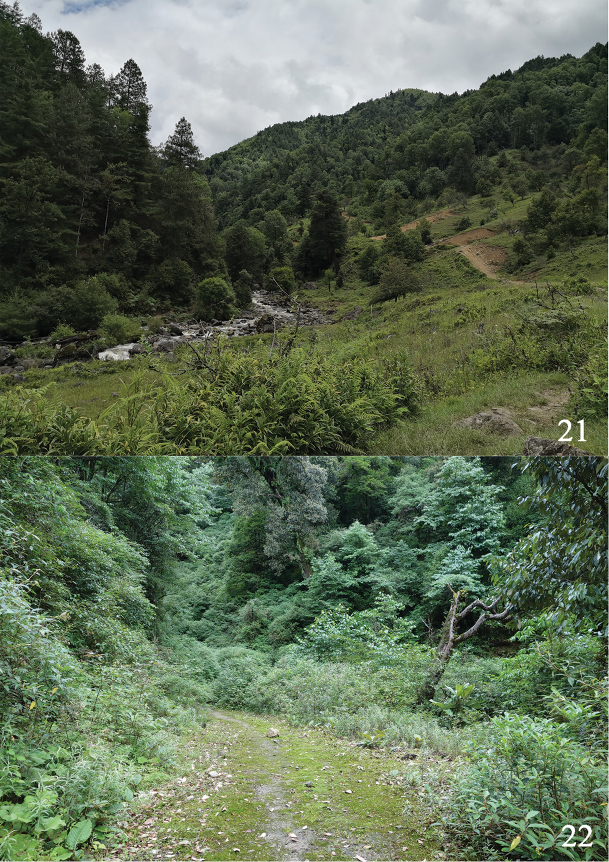
Habitats of *Psychostrophia micronymphidiaria* sp.nov. **21** near Shajiama Bridge, Weixi County **22** Yaojiaping, Lushui County.

**Figures 23, 24.**
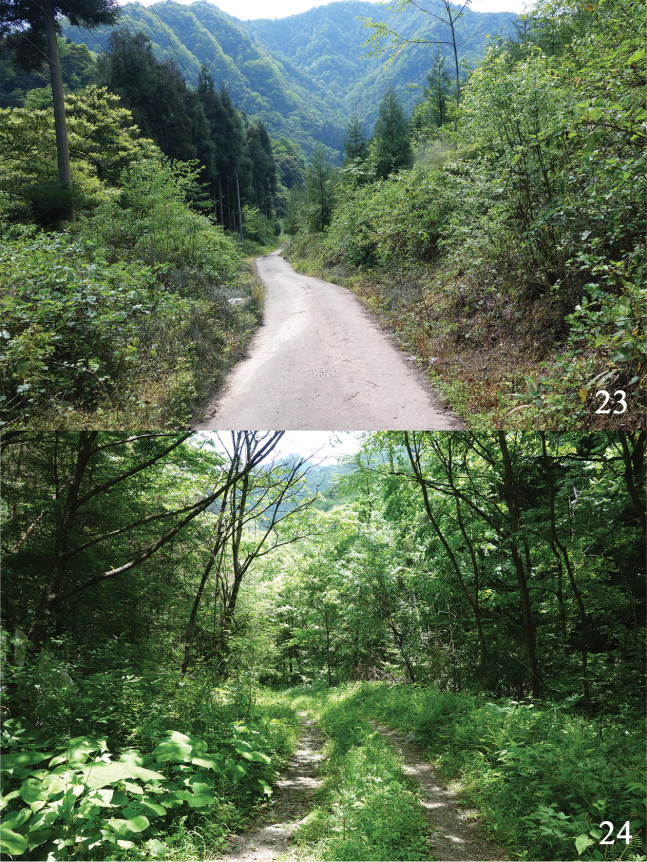
Habitats of *Psychostrophia nymphidiaria***23** Mt Niba, Yingjing County **22** Shennongjia, Yichang City.

**Figure 25.**
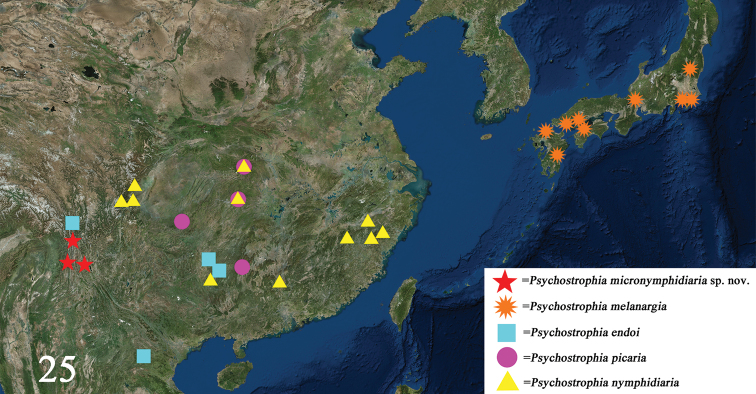
Distribution map of the genus *Psychostrophia*. Records of distribution are taken from Inoue (1992), Chou and Yuan (2001), Owada (2011), Huang et al. (2019), An Identification Guide of Japanese Moths Compiled by Everyone http://www.jpmoth.org, Shiiba Research Forest, Kyushu University http://www.forest.kyushu-u.ac.jp/miyazaki/index.php, and the present study.

##### Key to the genus *Psychostrophia* Butler, 1877

**Table:** 

1	Forewing from base to medial zone with two yellow or whitish areas, the basal one situated along discal cell, the outer one extending from costal region to postmedial region	***Psychostrophia melanargia***
–	Forewing from base to medial zone with only one pale white or whitish area	**2**
2	Pale area forming a slender band	**3**
–	Pale area forming a trapezoidal or triangular zone	**4**
3	Hindwing postmedial series comprised of a single transverse band	***Psychostrophia picaria***
–	Hindwing postmedial series comprised of several separated dots	***Psychostrophia endoi***
4	Forewing discal cell bar slender	***Psychostrophia micronymphidiaria* sp. nov.**
–	Forewing discal cell bar short and robust	***Psychostrophia nymphidiaria***

## Supplementary Material

XML Treatment for Psychostrophia

XML Treatment for Psychostrophia micronymphidiaria

XML Treatment for Psychostrophia nymphidiaria

XML Treatment for Psychostrophia picaria

XML Treatment for Psychostrophia endoi

XML Treatment for Psychostrophia melanargia
